# Nonadherence to Cervical Cancer Screening Guidelines in Commercially Insured US Adults, 2013-2021

**DOI:** 10.1001/jamanetworkopen.2025.48512

**Published:** 2025-12-10

**Authors:** Michelle B. Shin, Sarah Axeen, Allison M. Cole, X. Mona Guo, Jessica Y. Islam, Linda K. Ko, Connor R. Volpi, Rachel L. Winer, Jennifer Tsui

**Affiliations:** 1 Department of Child, Family, and Population Health Nursing, School of Nursing, University of Washington, Seattle; 2 Fred Hutch, University of Washington, Seattle Children’s Cancer Consortium, Seattle; 3 Department of Emergency Medicine, Keck School of Medicine, University of Southern California, Los Angeles; 4 Shaeffer Center for Health Policy and Economics, University of Southern California, Los Angeles; 5 Department of Family Medicine, School of Medicine, University of Washington, Seattle; 6 Division of Gynecologic Oncology, Department of Obstetrics and Gynecology, Keck School of Medicine, University of Southern California, Los Angeles; 7 Center for Immunization and Infection Research in Cancer, H. Lee Moffitt Cancer Center and Research Institute, Tampa, Florida; 8 Department of Cancer Epidemiology, H. Lee Moffitt Cancer Center and Research Institute, Tampa, Florida; 9 Department of Family Medicine, Keck School of Medicine, University of Southern California, Los Angeles; 10 Department of Population and Public Health Sciences, Keck School of Medicine, University of Southern California, Los Angeles; 11 Department of Epidemiology, Johns Hopkins Bloomberg School of Public Health, Baltimore, Maryland; 12 Department of Epidemiology, School of Public Health, University of Washington, Seattle; 13 Norris Comprehensive Cancer Center, University of Southern California, Los Angeles

## Abstract

This cohort study examines whether any sociodemographic characteristics or screening modalities are associated with adherence to guideline-based cervical cancer screening.

## Introduction

Cervical cancer screening guidelines in the US have evolved rapidly with newer screening modalities, including addition of quintennial cotesting in 2012 and primary HPV testing in 2018 to triennial cytology only,^1^ leading to widespread public confusion among patients and clincians.^2^ Evidence from countries like Australia, which shifted to human papillomavirus (HPV)–based screening only, shows that guideline confusion can lead to nonadherence—eg, overscreening and underscreening, defined as shorter and longer than guideline-based screening intervals, respectively.^3^ Overscreening can lead to unnecessary procedures and cost,^4^ while underscreening could worsen disparities in cancer burden among racial and ethnic minority individuals.^5^ However, the impact of these US guideline changes on adherence across modalities and populations remains understudied, raising concerns about widening gaps in care. Therefore, we examined cervical cancer screening guideline adherence among a nationally representative commercially insured cohort in the US to identify factors and modalities associated with nonadherence and test for interactions between race, ethnicity, and modality.

## Methods

In this cohort study, we analyzed 2013-2021 Optum Clinformatics, an anonymized longitudinal claims database covering approximately 13 million individuals annually with medical, laboratory, and procedure records. Inclusion and exclusion codes and definitions of adherence, overscreening, and underscreening appear in eTables 1 to 3 in Supplement 1.

Factors included modality, race and ethnicity,^6^ age, Charlson Comorbidity Index (CCI), and socioeconomic status (SES) variables (education, household net worth [HHN]). Primary HPV testing was excluded from modalities because it was not USPSTF-recommended until 2018. Multivariable logistic regression was used to estimate adjusted predicted probabilities (PPs) of overscreening and underscreening. Because adoption of screening modalities varies by race and ethnicity, which influences adherence,^5^ we tested their interaction using Wald χ^2^ test, adjusting for age, education, HHN, and CCI. Stata version 14 (StataCorp) was used for all analyses. Statistical significance was defined as *P* < .001. This study was deemed exempt from review and the requirement for informed consent by the institutional review boards of the University of Southern California and the University of Washington.

## Results

Among 670 003 eligible individuals, 315 249 (47.1%) received cytology only, and 353 754 (52.9%) received cotesting (Table). Only 49 941 (7.3%) were guideline adherent, while 412 777 (61.6%) were overscreened and 208 295 (31.1%) underscreened. The overall adjusted PP of overscreening was 89.4% (95% CI, 89.3%-89.5%) and underscreening was 81.0% (95% CI, 80.8%-81.1%) and broadly similar across racial and ethnic groups (Figure).

**Table.** Characteristics of Average-Risk Female Participants Who Received at Least 1 Cervical Cancer Screening, Overall and by Adherence Categories

Characteristic^b^	All eligible patients, No. (column %)	Patients, No. (row %)^a^				*P* value^c^
		Overscreening	Adherent		Underscreening	
Total	670 003 (100)	412 777 (61.6)		48 931 (7.3)	208 295 (31.1)	NA
Index screening modality						
Cytology only	315 249 (47.1)	184 607 (58.6)		39 916 (12.7)	90 726 (28.8)	<.001
Cotesting	354 754 (52.9)	228 170 (64.3)		9015 (2.5)	117 569 (33.1)	
Race and ethnicity^d^						
Hispanic	77 747 (11.6)	49 718 (63.9)		4726 (6.1)	23 303 (30.0)	<.001
Non-Hispanic Asian	38 870 (5.8)	23 532 (60.5)		2758 (7.1)	12 580 (32.4)	
Non-Hispanic Black	65 355 (9.8)	41 306 (63.2)		4231 (6.5)	19 818 (30.3)	
Non-Hispanic White	488 031 (72.8)	298 221 (61.1)		37 216 (7.6)	152 594 (31.3)	
Age, y						
30-39	176 933 (26.4)	120 511 (68.1)		13 052 (7.4)	43 370 (24.5)	<.001
40-49	201 716 (30.1)	132 533 (65.7)		14 286 (7.1)	54 897 (27.2)	
50-59	203 903 (30.4)	120 547 (59.1)		16 579 (8.1)	66 777 (32.7)	
60-64	87 451 (13.1)	39 186 (44.8)		5014 (5.7)	43 251 (49.5)	
Education level						
<Baccalaureate degree	479 229 (71.5)	285 793 (59.6)		36 328 (7.6)	157 198 (32.8)	<.001
≥Baccalaureate degree	190 774 (28.5)	126 984 (66.6)		12 693 (6.7)	51 097 (26.8)	
Household net worth						
<$250 000	290 530 (43.4)	170 679 (58.7)		21 750 (7.5)	98 101 (33.8)	<.001
$250 000-$499 999	115 782 (17.3)	71 234 (61.5)		9034 (7.8)	35 514 (30.7)	
≥500 000	263 691 (39.4)	170 864 (64.8)		18 147 (6.9)	74 680 (28.3)	
Charlson Comorbidity Index						
0-2	360 143 (53.8)	222 933 (61.9)		27 397 (7.6)	109 813 (30.5)	<.001
≥3	309 860 (46.2)	189 844 (61.3)		21 534 (6.9)	98 482 (31.8)	

Abbreviation: NA, not applicable.

^a^ The row percentages may not equal 100 due to rounding.

^b^ Characteristics were measured at the index screening. There were no missing data for index screening modality, age, and Charlson Comorbidity Index. Individuals whose status for race and ethnicity, education level, and household net worth were unknown were excluded.

^c^ *P* values indicate statistical significance from χ^2^ tests comparing the distribution of the screening adherence categories by each of the predictors of interest.

^d^ Race and ethnicity were constructed by Optum’s proprietary algorithm (E-Tech version 7.3), derived from individual- and area-level information.^6^

**Figure.**
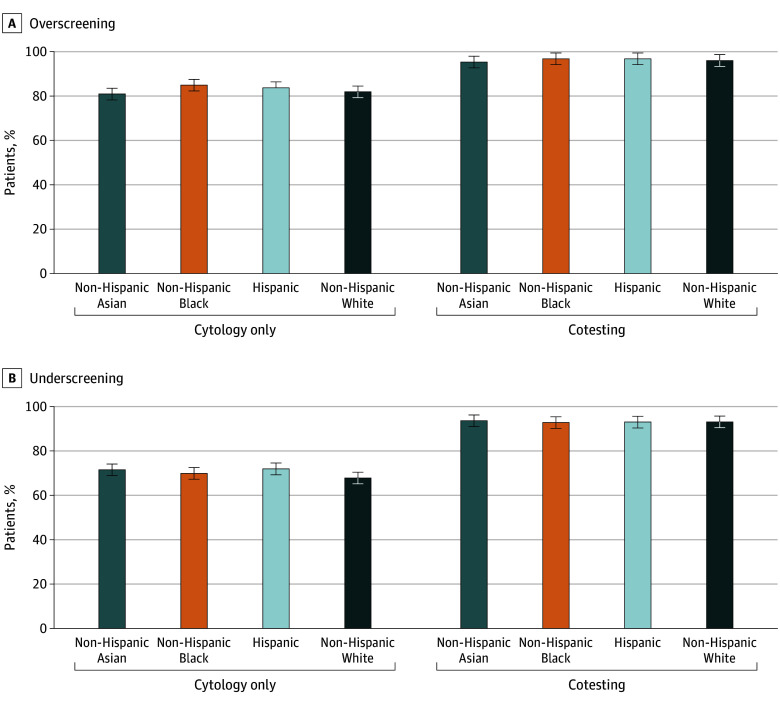
Predicted Probabilities of Overscreening and Underscreening by Index Screening Modality and Race and Ethnicity

Overscreening PP was higher among those screened with cotesting vs cytology only (96.2% [95% CI, 96.1%-96.2%] vs 82.4% [95% CI, 82.2%-82.5%]). Overscreening was highest among non-Hispanic (NH) Black (PP, 91.0% [95% CI, 90.8%-91.3%]), younger age, high CCI, and high SES (≥Baccalaureate degree: PP, 90.1% [95% CI, 89.9%-90.3%]; HHN ≥$500 000: PP, 89.8% [95% CI, 89.7%-90.0%]) groups. Underscreening PP was also higher with cotesting vs cytology only (93.1% [95% CI, 93.0%-93.2%] vs 68.7% [95% CI, 68.4%-68.9%]) and highest among NH Asian (PP, 82.7% [95% CI, 82.1%-83.3%]), older age, high CCI, and low SES (<Baccalaureate degree: PP, 81.3% [95% CI, 81.1%-81.5%]; HHN <$250 000: PP, 82.8% [95% CI, 82.6%-83.0%]) groups.

Interaction analyses showed that overscreening and underscreening PP were higher among those screened with cotesting vs cytology across all racial and ethnic groups. Overscreening PP was the highest among NH Black (96.8% [95% CI, 96.6%-97.1%] vs 84.9% [95% CI, 84.5%-85.4%]) and lowest among NH Asian (95.3% [95% CI, 95.0%-95.7%] vs 80.9% [95% CI, 80.2%-81.7%]) participants. Underscreening PP was the highest in NH Asian patients (PP, 93.6%, [95% CI, 93.1%-94.1%]) among individuals screened with cotesting, and in Hispanic patients (PP, 71.9% [95% CI, 71.1%-72.6%]) among those screened with cytology only.

## Discussion

Consistent with prior work, we observed low adherence (7.3%) to cervical cancer screening guidelines among the commercially insured US population despite stable coverage,^4,5^ likely reflecting guideline confusion among patients, clinicians, and health systems.^1,2^ More evidence-based strategies are needed to expand capacity for guideline-adherent screening, reduce overscreening, and align payer and health system incentives, particularly as new modalities, such as HPV self-sampling, emerge. Findings also suggest that the impact of newer modalities on screening quality varies by race and ethnicity, underscoring the need for community-tailored interventions to avoid widening disparities. Key limitations include exclusion of uninsured populations, potential coding errors, and imperfect race and ethnicity classification, which may affect generalizability.

Guideline adherence was less than 10%, and screening modality was strongly associated with nonadherence. Targeted strategies are needed to improve adherence, de-implement unnecessary care, and address disparities in adoption of evolving screening guidelines.

## Notes

Supplement 1. **eTable 1.** Cytology and HPV Testing Codes **eTable 2.** Exclusion Codes **eTable 3.** Definitions of Adherence, Overscreening, and Underscreening Adapted From the 2012 American College of Obstetricians and Gynecologists Cervical Cancer Screening Guideline for Ages 30 to 64 Years
eReferences.

Supplement 2.
Data Sharing Statement
