# Supplementary material for: Nonadherence to Cervical Cancer Screening Guidelines in Commercially Insured US Adults, 2013-2021
Source: JAMA Netw Open. 2025 Dec 10;8(12):e2548512. doi: 10.1001/jamanetworkopen.2025.48512 (PMC12696592; doi:10.1001/jamanetworkopen.2025.48512)
Supplement: Supplement 1. — eTable 1. Cytology and HPV Testing Codes eTable 2. Exclusion Codes eTable 3. Definitions of Adherence, Overscreening, and Underscreening Adapted From the 2012 American College of Obstetricians and Gynecologists Cervical Cancer Screening Guideline for Ages 30 to 64 Years eReferences. [file jamanetwopen-e2548512-s001.pdf]

Supplemental Online Content

Shin MB, Axeen S, Cole AM, et al. Nonadherence to Cervical cancer screening guidelines in commercially insured US Adults, 2013-2021. *JAMA Netw Open*. 2025;8(12):e2548512. doi:10.1001/jamanetworkopen.2025.48512

eTable 1. Cytology and HPV Testing Codes
eTable 2. Exclusion Codes
eTable 3. Definitions of Adherence, Overscreening, and Underscreening Adapted From the 2012 American College of Obstetricians and Gynecologists Cervical Cancer Screening Guideline for Ages 30 to 64 Years
eReferences.

This supplemental material has been provided by the authors to give readers additional information about their work.

© 2025 Shin MB et al. *JAMA Network Open*.

eTable 1. Cytology and HPV Testing Codes

Type	Code	Description
**Cytology**		
CPT	88141	Cytopathology, cervical or vaginal (any reporting system), requiring interpretation by physician
CPT	88142	Cytopathology, cervical or vaginal (any reporting system), collected in preservative fluid, automated thin layer preparation; manual screening under physician supervision
CPT	88143	Cytopathology, cervical or vaginal (any reporting system), collected in preservative fluid, automated thin layer preparation; with manual screening and rescreening under physician supervision
CPT	88147	Cytopathology smears, cervical or vaginal; screening by automated system under physician supervision
CPT	88148	Cytopathology smears, cervical or vaginal; screening by automated system with manual rescreening under physician supervision
CPT	88150	Cytopathology, slides, cervical or vaginal; manual screening under physician supervision
CPT	88152	Cytopathology, slides, cervical or vaginal; with manual screening and computer-assisted rescreening under physician supervision
CPT	88153	Cytopathology, slides, cervical or vaginal; with manual screening and rescreening under physician supervision
CPT	88154	Cytopathology, slides, cervical or vaginal; with manual screening and computer-assisted rescreening using cell selection and review under physician supervision
CPT	88155	C ytopathology, slides, cervical or vaginal, definitive hormonal evaluation (e.g., maturation index, karyopyknotic index, estrogenic index) (List separately in addition to code[s] for other technical and interpretation services)
CPT	88164	Cytopathology, slides, cervical or vaginal (the Bethesda System); manual screening under physician supervision
CPT	88165	Cytopathology, slides, cervical or vaginal (the Bethesda System); with manual screening and rescreening under physician supervision
CPT	88166	Cytopathology, slides, cervical or vaginal (the Bethesda System); with manual screening and computer-assisted rescreening under physician supervision
CPT	88167	Cytopathology, slides, cervical or vaginal (the Bethesda System); with manual screening and computer-assisted rescreening using cell selection and review under physician supervision
CPT	88174	Cytopathology, cervical or vaginal (any reporting system), collected in preservative fluid, automated thin layer preparation; screening by automated system, under physician supervision
CPT	88175	Cytopathology, cervical or vaginal (any reporting system), collected in preservative fluid, automated thin layer preparation; with screening by automated system and manual rescreening or review, under physician supervision
HCPCS	G0101	Cervical or vaginal cancer screening; pelvic and clinical breast examination
HCPCS	G0123	Screening cytopathology, cervical or vaginal (any reporting system, collected in preservative fluid, automated thin layer preparation, screening by cytotechnologist under physician supervision
HCPCS	G0124	Screening cytopathology, cervical or vaginal (any reporting system), collected in preservative fluid, automated thin layer preparation, requiring interpretation by physician
HCPCS	G0141	Screening cytopathology smears, cervical or vaginal, performed by automated system, with manual rescreening, requiring interpretation by physician
HCPCS	G0143	Screening cytopathology, cervical or vaginal (any reporting system), collected in preservative fluid, automated thin layer preparation, with manual screening and rescreening by cytotechnologist under physician supervision

© 2025 Shin MB et al. *JAMA Network Open*.

HCP CS	G014 4	Screening cytopathology, cervical or vaginal (any reporting system), collected in preservative fluid, automated thin layer preparation, with screening by automated system, under physician supervision
HCP CS	G014 5	Screening cytopathology, cervical or vaginal (any reporting system), collected in preservative fluid, automated thin layer preparation, with screening by automated system and manual rescreening under physician supervision
HCP CS	G014 7	Screening cytopathology smears, cervical or vaginal, performed by automated system under physician supervision
HCP CS	G014 8	Screening cytopathology smears, cervical or vaginal, performed by automated system with manual rescreening
HCP CS	P300 0	Screening Papanicolaou smear, cervical or vaginal, up to three smears, by technician under physician supervision
HCP CS	P300 1	Screening Papanicolaou smear, cervical or vaginal, up to three smears, requiring interpretation by physician
HCP CS	Q009 1	Screening Papanicolaou smear; obtaining, preparing and conveyance of cervical or vaginal smear to laboratory
ICD-9	91.46	Microscopic examination of specimen from female genital tract, cell block and Papanicolaou smear
ICD-9	V76.2	Routine cervical Papanicolaou smear
ICD-10	Z12.4	Encounter for screening pap smear for malignant neoplasm of cervix
**HPV testing**		
CPT	0500 T	Infectious agent detection by nucleic acid (DNA or RNA), human papillomavirus (HPV) for five or more separately reported high-risk HPV types (eg, 16, 18, 31, 33, 35, 39, 45, 51, 52, 56, 58, 59, 68) (i.e., genotyping)
CPT	8762 0	Infectious agent detection by nucleic acid (DNA or RNA); papillomavirus, human, direct probe technique
CPT	8762 1	Infectious agent detection by nucleic acid (DNA or RNA); papillomavirus, human, amplified probe technique
CPT	8762 2	Infectious agent detection by nucleic acid (DNA or RNA); papillomavirus, human, quantification
CPT	8762 4	Infectious agent detection by nucleic acid (DNA or RNA); HPV, high-risk types (e.g., 16, 18, 31, 33, 35, 39, 45, 51, 52, 56, 58, 59, 68)
CPT	8762 5	Infectious agent detection by nucleic acid (DNA or RNA); HPV, types 16 and 18 only, includes type 45, if performed
HCP CS	G047 6	Infectious agent detection by nucleic acid (DNA or RNA); HPV, high-risk types (e.g., 16, 18, 31, 33, 35, 39, 45, 51, 52, 56, 58, 59, 68) for cervical cancer screening, must be performed in addition to pap test
ICD-9	V73.8 1	Special screening examination for Human papillomavirus (HPV)
ICD-10	Z11.5 1	Encounter for screening for human papillomavirus (HPV)

© 2025 Shin MB et al. *JAMA Network Open*.

eTable 2. Exclusion Codes

Type	Code	Description
CPT	51925	Closure of vesicouterine fistula; with hysterectomy
CPT	56308	laparoscopy, surgical; with vaginal hysterectomy with or without removal of tube[s], with or without removal of ovary[s] [laproscopic assisted vaginal hysterectomy]
CPT	57540	Excision of cervical stump, abdominal approach
CPT	57545	Excision of cervical stump, abdominal approach; with pelvic floor repair
CPT	57550	Excision of cervical stump, vaginal approach
CPT	57555	Excision of cervical stump, vaginal approach; with anterior and/or posterior repair
CPT	57556	Excision of cervical stump, vaginal approach; with repair of enterocele
CPT	58150	Total abdominal hysterectomy (corpus and cervix), with or without removal of tube(s), with or without removal of ovary(s)
CPT	58152	Total abdominal hysterectomy (corpus and cervix), with or without removal of tube(s), with or without removal of ovary(s)
CPT	58200	Total abdominal hysterectomy, including partial vaginectomy, with para-aortic and pelvic lymph node sampling, with or without removal of tube(s), with or without removal of ovary(s)
CPT	58210	Radical abdominal hysterectomy, with bilateral total pelvic lymphadenectomy and para-aortic lymph node sampling (biopsy), with or without removal of tube(s), with or without removal of ovary(s)
CPT	58240	Pelvic exenteration for gynecologic malignancy, with total abdominal hysterectomy or cervicectomy, with or without removal of tube(s), with or without removal of ovary(s), with removal of bladder and ureteral transplantations, and/or abdominoperineal resection of rectum and colon and colostomy, or any combination thereof
CPT	58260	Vaginal hysterectomy, for uterus 250 g or less
CPT	58262	Vaginal hysterectomy, for uterus 250 g or less; with removal of tube(s), and/or ovary(s)
CPT	58263	Vaginal hysterectomy, for uterus 250 g or less; with removal of tube(s), and/or ovary(s), with repair of enterocele
CPT	58267	Vaginal hysterectomy, for uterus 250 g or less; with colpo-urethrocystopexy (Marshall-Marchetti-Krantz type, Pereyra type) with or without endoscopic control
CPT	58270	Vaginal hysterectomy, for uterus 250 g or less; with repair of enterocele
CPT	58275	Vaginal hysterectomy, with total or partial vaginectomy
CPT	58280	Vaginal hysterectomy, with total or partial vaginectomy; with repair of enterocele
CPT	58285	Vaginal hysterectomy, radical (Schauta type operation)
CPT	58290	Vaginal hysterectomy, for uterus greater than 250 g
CPT	58291	Vaginal hysterectomy, for uterus greater than 250 g; with removal of tube(s) and/or ovary(s)
CPT	58292	Vaginal hysterectomy, for uterus greater than 250 g; with removal of tube(s) and/or ovary(s), with repair of enterocele
CPT	58293	Vaginal hysterectomy, for uterus greater than 250 g; with colpo-urethrocystopexy (Marshall-Marchetti-Krantz type, Pereyra type) with or without endoscopic control
CPT	58294	Vaginal hysterectomy, for uterus greater than 250 g; with repair of enterocele

© 2025 Shin MB et al. *JAMA Network Open*.

CPT	56308	Laparoscopy, surgical; with vaginal hysterectomy with or without removal of tube[s], with or without removal of ovary[s] [laproscopic assisted vaginal hysterectomy]
CPT	58548	Laparoscopy, surgical, with radical hysterectomy, with bilateral total pelvic lymphadenectomy and para-aortic lymph node sampling (biopsy), with removal of tube(s) and ovary(s), if performed
CPT	58550	Laparoscopy, surgical, with vaginal hysterectomy, for uterus 250 g or less
CPT	58552	Laparoscopy, surgical, with vaginal hysterectomy, for uterus 250 g or less; with removal of tube(s) and/or ovary(s)
CPT	58553	Laparoscopy, surgical, with vaginal hysterectomy, for uterus greater than 250 g
CPT	58554	Laparoscopy, surgical, with vaginal hysterectomy, for uterus greater than 250 g; with removal of tube(s) and/or ovary(s)
CPT	58570	Laparoscopy, surgical, with total hysterectomy, for uterus 250 g or less
CPT	58571	Laparoscopy, surgical, with total hysterectomy, for uterus 250 g or less; with removal of tube(s) and/or ovary(s)
CPT	58572	Laparoscopy, surgical, with total hysterectomy, for uterus greater than 250 g
CPT	58573	Laparoscopy, surgical, with total hysterectomy, for uterus greater than 250 g; with removal of tube(s) and/or ovary(s)
CPT	58951	Resection (initial) of ovarian, tubal or primary peritoneal malignancy with bilateral salpingo-oophorectomy and omentectomy; with total abdominal hysterectomy, pelvic and limited para-aortic lymphadenectomy
CPT	59525	Subtotal or total hysterectomy after cesarean delivery (List separately in addition to code for primary procedure)
CPT	58953	Bilateral salpingo-oophorectomy with omentectomy, total abdominal hysterectomy and radical dissection for debulking
CPT	58954	Bilateral salpingo-oophorectomy with omentectomy, total abdominal hysterectomy and radical dissection for debulking; with pelvic lymphadenectomy and limited para-aortic lymphadenectomy
CPT	58956	Bilateral salpingo-oophorectomy with total omentectomy, total abdominal hysterectomy for malignancy
CPT	59135	Surgical treatment of ectopic pregnancy; interstitial, uterine pregnancy requiring total hysterectomy
CPT	58575	Laparoscopy, surgical, total hysterectomy for resection of malignancy (tumor debulking), with omentectomy including salpingo-oophorectomy, unilateral or bilateral, when performed
CPT	59525	Subtotal or total hysterectomy after cesarean delivery (List separately in addition to code for primary procedure)
CPT	57522	Conization of cervix, with or without fulguration, with or without dilation and curettage, with or without repair; loop electrode excision
CPT	57460	Colposcopy of the cervix including upper/adjacent vagina; with loop electrode biopsy(s) of the cervix
CPT	57461	Colposcopy of the cervix including upper/adjacent vagina; with loop electrode conization of the cervix
CPT	57510	Cautery of cervix; electro or thermal
CPT	57511	Cautery of cervix; cryocautery, initial or repeat
CPT	57510	Cautery of cervix; electro or thermal
CPT	57513	Cautery of cervix; laser ablation
CPT	57420	Colposcopy of the entire vagina, with cervix if present
CPT	57421	Colposcopy of the entire vagina, with cervix if present; with biopsy(s) of vagina/cervix
CPT	57505	Endocervical curettage (not done as part of a dilation and curettage)

© 2025 Shin MB et al. *JAMA Network Open*.

CPT	57500	Biopsy of cervix, single or multiple, or local excision of lesion, with or without fulguration (separate procedure)
CPT	57452	Colposcopy of the cervix including upper/adjacent vagina
CPT	57454	Colposcopy of the cervix including upper/adjacent vagina with biopsy(s) of the cervix and endocervical curettage
CPT	57455	Colposcopy of the cervix including upper/adjacent vagina with biopsy(s) of the cervix
CPT	57456	Colposcopy of the cervix including upper/adjacent vagina with endocervical curettage
CPT	57460	Colposcopy of the cervix including upper/adjacent vagina with loop electrode biopsy(s) of the cervix
CPT	57461	Colposcopy of the cervix including upper/adjacent vagina with loop electrode conization of the cervix
CPT	57520	Conization of cervix, with or without fulguration, with or without dilation and curettage, with or without repair; cold knife or laser
ICD-10	C53	Malignant neoplasm of cervix uteri
ICD-10	C53.0	Malignant neoplasm of endocervix
ICD-10	C53.1	Malignant neoplasm of exocervix
ICD-10	C53.8	Malignant neoplasm of overlapping sites of cervix uter
ICD-10	C53.9	Malignant neoplasm of cervix uteri, unspecified
ICD-10	Z85.41	Personal history of malignant neoplasm of cervix uteri
ICD-10	Z87.410	Personal history of cervical dysplasia
ICD-10-CM	Z90.710	Acquired absence of both cervix and uterus
ICD-10-CM	Z90.712	Acquired absence of cervix with remaining uterus
ICD-10-CM	Z12.72	Encounter for screening for malignant neoplasm of vagina
ICD-10-PCS	0UTC0ZZ	Resection of Cervix, Open Approach
ICD-10-PCS	0UTC4ZZ	Resection of Cervix, Percutaneous Endoscopic Approach
ICD-10-PCS	0UTC7ZZ	Resection of Cervix, Via Natural or Artificial Opening
ICD-10-PCS	0UTC8ZZ	Resection of Cervix, Via Natural or Artificial Opening Endoscopic
ICD-10-PCS	0UT90ZZ	Resection of Uterus, Open Approach
ICD-10-PCS	0UT94ZZ	Resection of Uterus, Percutaneous Endoscopic Approach
ICD-10-PCS	0UT97ZZ	Resection of Uterus, Via Natural or Artificial Opening
ICD-10-PCS	0UT98ZZ	Resection of Uterus, Via Natural or Artificial Opening Endoscopic
ICD-10-PCS	0UT9FZZ	Resection of Uterus, Via Natural or Artificial Opening With Percutaneous Endoscopic Assistance
ICD-10-PCS	0U5C0ZZ	Destruction of Cervix, Open Approach

© 2025 Shin MB et al. *JAMA Network Open*.

ICD-10-PCS	0U5C3ZZ	Destruction of Cervix, Percutaneous Approach
ICD-10-PCS	0U5C4ZZ	Destruction of Cervix, Percutaneous Endoscopic Approach
ICD-10-PCS	0U5C7ZZ	Destruction of Cervix, Via Natural or Artificial Opening
ICD-10-PCS	0U5C8ZZ	Destruction of Cervix, Via Natural or Artificial Opening Endoscopic
ICD-10-PCS	0UBC0ZX	Excision of Cervix, Open Approach, Diagnostic
ICD-10-PCS	0UBC3ZX	Excision of Cervix, Percutaneous Approach, Diagnostic
ICD-10-PCS	0UBC4ZX	Excision of Cervix, Percutaneous Endoscopic Approach, Diagnostic
ICD-10-PCS	0UBC7ZX	Excision of Cervix, Via Natural or Artificial Opening, Diagnostic
ICD-10-PCS	0UBC8ZX	Excision of Cervix, Via Natural or Artificial Opening Endoscopic, Diagnostic
ICD-9-CM	67.32	Destruction of lesion of cervix by cauterization
ICD-9-CM	67.33	Destruction of lesion of cervix by cryosurgery
ICD-9-CM	180	Malignant neoplasm of cervix uteri
ICD-9-CM	180	Malignant neoplasm of endocervix
ICD-9-CM	180.1	Malignant neoplasm of exocervix
ICD-9-CM	180.8	Malignant neoplasm of other specified sites of cervix
ICD-9-CM	180.9	Malignant neoplasm of cervix uteri, unspecified site
ICD-9-CM	V10.41	Personal history of malignant neoplasm of cervix uteri
ICD-9-CM	V13.22	Personal history of cervical dysplasia
ICD-9-CM	V72.32	Encounter for Papanicolaou cervical smear to confirm findings of recent normal smear following initial abnormal smear
ICD-9-CM	V88.01	Acquired absence of both cervix and uterus
ICD-9-CM	V88.03	Acquired absence of cervix with remaining uterus
ICD-9-PCS	67.11	Endocervical biopsy
ICD-9-PCS	67.12	Other cervical biopsy
ICD-9-PCS	67.19	Other diagnostic procedures on cervix
ICD-9-PCS	67.2	Conization of cervix
ICD-9-PCS	68.41	Laparoscopic total abdominal hysterectomy
ICD-9-PCS	68.49	Other and unspecified total abdominal hysterectomy
ICD-9-PCS	68.51	Laparoscopically assisted vaginal hysterectomy (LAVH)
ICD-9-PCS	68.59	Other and unspecified vaginal hysterectomy
ICD-9-PCS	68.61	Laparoscopic radical abdominal hysterectomy

© 2025 Shin MB et al. *JAMA Network Open*.

ICD-9-PCS	68.69	Other and unspecified radical abdominal hysterectomy
ICD-9-PCS	68.71	Laparoscopic radical vaginal hysterectomy [LRVH]
ICD-9-PCS	68.79	Other and unspecified radical vaginal hysterectomy
ICD-9-PCS	68.9	Other and unspecified hysterectomy

© 2025 Shin MB et al. *JAMA Network Open*.

eTable 3. Definitions of Adherence, Overscreening, and Underscreening Adapted From the 2012 American College of Obstetricians and Gynecologists Cervical Cancer Screening Guideline for Ages 30 to 64 Years

Adherent	Co-testing*: 4.5-5.5 years†<br>Cytology-only: 2.5-3.5 years
Over-screening	Co-testing: <4.5 years<br>Cytology-only: <2.5 years
Under-screening	Co-testing: >5.5 years<br>Cytology-only: >3.5 years<br>Lack of subsequent screening

*Co-testing was defined as the occurrence of cytology and HPV testing within 30 days

†Primary HPV testing was not introduced until 2016 in the ACOG guidelines, 2018 in the United States Preventive Services Task Force guidelines, and 2020 in the American Cancer Society guidelines[1-3]

© 2025 Shin MB et al. *JAMA Network Open*.

Supplemental References

1. ACOG Practice Bulletin Number 131: Screening for cervical cancer. *Obstetrics and gynecology*. Nov 2012;120(5):1222-38. doi:10.1097/aog.0b013e318277c92a
2. Moyer VA. Screening for cervical cancer: U.S. Preventive Services Task Force recommendation statement. *Ann Intern Med*. Jun 19 2012;156(12):880-91, w312. doi:10.7326/0003-4819-156-12-201206190-00424
3. Saslow D, Solomon D, Lawson HW, et al. American Cancer Society, American Society for Colposcopy and Cervical Pathology, and American Society for Clinical Pathology screening guidelines for the prevention and early detection of cervical cancer. https://doi.org/10.3322/caac.21139. *CA: a cancer journal for clinicians*. 2012/05/01 2012;62(3):147-172. doi:https://doi.org/10.3322/caac.21139

© 2025 Shin MB et al. *JAMA Network Open*.
