# Supplementary material for: Nonadherence to Cervical Cancer Screening Guidelines in Commercially Insured US Adults, 2013-2021
Source: JAMA Netw Open. 2025 Dec 10;8(12):e2548512. doi: 10.1001/jamanetworkopen.2025.48512 (PMC12696592; doi:10.1001/jamanetworkopen.2025.48512)
Supplement: Supplement 2. — Data Sharing Statement [file jamanetwopen-e2548512-s002.pdf]

Data Sharing Statement

Shin. Nonadherence to Cervical Cancer Screening Guidelines in Commercially Insured US Adults, 2013-2021. *JAMA Netw Open*. Published December 10, 2025. doi:10.1001/jamanetworkopen.2025.48512

Data
Data available: No

Additional Information
Explanation for why data not available: The data used in this study were collected by a third party and are not publicly available due to restrictions on data sharing.
